# Patients with questionable penicillin (beta-lactam) allergy: Causes and solutions 

**DOI:** 10.5414/ALX02310E

**Published:** 2022-02-01

**Authors:** Knut Brockow, Gerda Wurpts, Axel Trautmann

**Affiliations:** 1 Department of Dermatology and Allergology Biederstein, Faculty of Medicine, Technical University of Munich, Munich,; 2 Clinic for Dermatology and Allergology, Aachen Comprehensive Allergy Center (ACAC), University Hospital of RWTH Aachen, and; 3 Department of Dermatology and Allergology, Allergy Center Mainfranken, University Hospital Würzburg, Germany

**Keywords:** β-lactam-antibiotics, penicillin, allergy, β-lactam allergy, labeling, delabeling, allergy diagnostics

## Abstract

Background: In Europe, North America, and Australia, 5% to 10% of the population are now classified as penicillin (β-lactam) allergic. Only ~ 10% of these questionable diagnoses, mostly made in childhood, can be confirmed by allergy diagnostics. Materials and methods: The aim of this review is to show causes and consequences as well as recommendations for dealing with the often questionable diagnosis of penicillin (β-lactam) allergy (BLA). Results: An incorrect BLA diagnosis may negatively impact antibiotic treatment needed in the future, by using a less effective antibiotic or using a broad-spectrum antibiotic, for example, further exacerbating the problem of increasing antibiotic resistance. Accordingly, there is growing pressure from antibiotic stewardship programs to critically challenge the BLA diagnosis. Conservatively, a suspected BLA is reviewed by an allergist using medical history, skin testing, laboratory testing, and provocation. This clarification is costly and is not remunerated in the German health care system; that is the reason why this testing is only offered in a few specialized clinics and practically not at all in general practice. In view of thousands of affected patients, additional strategies are needed to treat patients with a low risk of hypersensitivity reaction despite suspected allergy with a β-lactam antibiotic. In recent years, various methods have been proposed to eliminate suspected allergy as promptly as possible and directly before necessary treatment with a β-lactam antibiotic, including standardized history (also in the form of an algorithm), skin test with immediate reading after 15 minutes, or administration of a small test dose. Investigations of small case series and also multi-center studies to date have yielded promising results in terms of feasibility and safety. Conclusion: Of the large number of patients with (questionable) BLA, most have never been tested and – if antibiotic treatment becomes necessary – simply receive an alternative antibiotic. The diagnosis of BLA therefore requires new approaches besides classical allergy testing to critically question BLA.

## Introduction 

β-lactam antibiotics (BL) are the first-line treatment for many bacterial infections [1, 2, 3]. However, they are also the most common triggers of drug allergies and a frequent cause of fatal drug anaphylaxis [4, 5]. BL allergy (BLA) can trigger all four types of hypersensitivity reactions described by Coombs and Gell, of which only immediate-type anaphylaxis and T-cell-mediated drug exanthema are common and thus most significant [6]. A vague possibility of a BLA in a patient often leads to a suspicion of having BLA with documentation in an allergy passport and subsequent avoidance of BL [7]. It is estimated that ~ 5 – 10 % of the general population and up to 19 % of hospitalized patients are classified as BL allergic [2, 8, 9, 10]. Other publications report frequencies of up to 25% [3]. In most of these patients classified as BLA, the suspected allergy proves to be unfounded. The diagnosis can only be confirmed in a maximum of 10% of these patients by allergological tests, including drug provocation tests [9, 11]. In particular, allergies can very rarely be detected in children or in adults with childhood reactions and administration of BL in the context of infectious diseases [12, 13]. Many patients report their supposed “penicillin allergy” in childhood without clearly remembering or understanding their reaction, and often this suspicion is uncritically transferred to an allergy passport. Some important causes of misdiagnosis of BLA are shown in Table 1. Patients with a BLA diagnosis usually do not receive BL in the future, but instead receive second-line alternative antibiotics [14]. Antibiotic stewardship programs are gaining importance in health policy. They aim to improve treatment outcomes and prevent the emergence and spread of multidrug-resistant germs. Patients with a BLA label do not only receive inappropriate less effective antibiotics, but also receive more broad-spectrum antibiotics; in addition, they may experience delayed initiation of antibiotic therapy and increased surgical wound infections (Table 2) [1, 2, 9, 14, 15]. The alternative non-BLA antibiotics also result in higher treatment costs, longer hospital length of stay, higher re-admission rates, and a higher prevalence of antibiotic-resistant bacteria than in patients without a BLA label [1, 2, 16, 17]. This scenario could be avoided if the diagnosis of BLA could be critically questioned and, if necessary, refuted directly before treatment is started. This procedure is referred to in the English-language medical literature as “delabeling”. The stated necessity here is, on the one hand, to identify patients at risk for a renewed reaction, particularly a severe one, and on the other hand, to remove incorrect BLA labeling and thereby allow the use of BL antibiotics with acceptable safety [18]. 

## Central importance of the patient’s medical history 

Taking a medical history remains the most important method in allergology, not only to establish a first tentative diagnosis, but especially to estimate the probability of a future (severe) allergic reaction to the renewed use of a BL antibiotic, which may vary considerably in individual cases. The diagnosis of BLA and thus the (lifelong) labeling of the affected person as having BLA (mostly “penicillin allergy”) takes place in many cases already in childhood, often after (unnecessary) BL administration in the context of a viral infection of the upper respiratory tract. In ~ 75% of children with “penicillin allergy” the diagnosis is given before the age of 3 years [19]. However, most children do not have a typical medical history consistent with BLA [20]. In many cases, the clinical symptoms do not match an allergic reaction, e.g., in the case of nausea, vomiting, or diarrhea alone (Table 1). In individual cases, even a “family history of penicillin allergy” is seen as an indication of allergy [19]. Various acute skin reactions (the classic “rash”) are the most common causes of suspected BLA in childhood [21]. Most of these skin reactions attributed to BL are actually expressions of the infectious disease itself, e.g., acute urticaria or maculopapular exanthema triggered by the febrile infectious disease itself [12, 13]. 

Allergists naturally bring the most experience to correctly assess a questionable BLA and, in low-risk cases, remove the label BLA directly without prior testing. In this context, the description of the symptomatology experienced, the temporal occurrence, and the course of a possible reaction can provide important clues to estimate the likelihood of a (severe) allergic reaction to the re-introduction of a BL antibiotic (Table 3) [22, 23]. Sometimes, however, important details are missing from the history, so that even an experienced allergist may be forced to leave the diagnosis of BLA as a precautionary measure [24]. 

The capacity of specialized allergological care in Germany is limited and far from sufficient to test the many thousands of patients with (in > 90% incorrect) diagnosis of BLA. If at the same time, in the context of antibiotic stewardship programs, “delabeling” is declared to be an important task of the health care system, this implies a call to the entire medical profession. Lin et al. [25] reported on a successful BLA delabeling program for hospitalized patients in the Netherlands conducted by general practitioners. The intervention included an information campaign for all participating physicians, the issuance of pocket-sized reminder cards, and a corresponding link in the electronic medical record to motivate physicians to perform the required screening. A treatment pathway was successfully established in a U.S. emergency department with administration of test doses by nurses after physician risk assessment and subsequent monitoring [26]. Increasingly, pharmacologists are also being involved in the BLA assessment process [27]. 

## Verification of a BLA diagnosis 

There are several options for managing patients who have been classified as BL allergic at some point (often many years ago) (Table 4). The choice of a particular procedure depends on both the patient’s medical history and the availability of specialized allergy care (allergy testing including provocation). On one end of the spectrum, there are patients with a medical history indicative of a high likelihood of BLA (Table 3) who should be tested primarily by an allergist: skin prick, intradermal +/– patch tests, specific IgE +/– basophil activation test, followed by oral provocation test. On the other hand, there are cases with a very questionable BLA diagnosis that can already be largely excluded based on their medical history; they could be treated directly with BL. In between are patients with incomplete medical history or mild reactions (e.g., maculopapular exanthema) for whom delabeling without testing implies a certain residual risk, which, however, may be justifiable in individual cases. Above all, it is important to largely exclude severe reactions. The strengths, limitations, and differing evidence for the different procedures will be briefly addressed below. 

## Allergological routine diagnostics including provocation testing 

The usual BL allergy test in patients with a history of high risk of BLA requires a detailed, often standardised history, a skin test (skin prick test, intradermal test, and/or patch test), a laboratory test (specific IgE, possibly basophil activation test, rarely lymphocyte transformation test or ELISPOT test), and, most importantly, an oral provocation test with fractionated administration of BL [28]. The latter still remains the most important test method for confirmation, and even if a BLA to a preparation (e.g., amoxicillin) is detected, tolerant BLA (mostly all non-aminopenicillins and non-cephalosporins) and cross-reactivities can be identified [10]. 

## Skin testing without provocation testing 

Positive skin testing at recommended concentrations for one or more BLs is an important clue and, together with the appropriate medical history, can demonstrate allergy [28]. Negative skin tests at recommended concentrations and skin test series can minimize the risk of a reaction to a BL. Romano et al. [29] reported a loss of skin test reactivity in 10% of patients with BLA per year. In these patients < 1% will experience recurrence of immunologic reactions even after multiple administrations of BL [12, 30]. Non-allergists have also successfully used skin testing to rule out (severe) reactions before administration of BL in inpatient and outpatient settings [31, 32]. 

## Provocation testing without skin testing 

A less resource-intensive method uses direct oral provocation in patients with a history of low risk for non-severe BLA. The direct oral provocation test has also been performed outside of allergy centers [33, 34, 35, 36, 37, 38, 39]. Such testing is useful when 1) there is a low risk of triggering a reaction, and 2) a severe reaction can be largely excluded. Such a situation exists, for example, in children who very frequently develop infection-related maculopapular exanthema in (non-causal) association with the administration of BL. In this population, skin testing was not informative for the recurrence of exanthema after provocation; when reactions occurred, they were all mild [13]. 

## Standardized questions (consideration of the medical history alone) 

In more than 90% of the patients examined, the reported and possibly also documented BLA diagnosis cannot be confirmed in an allergy test (including provocation). In some of these cases, a sufficiently reliable assessment of the risk is possible on the basis of a (standardized) medical history alone, e.g., in the case of only gastrointestinal complaints after BL administration, a “mild rash” in childhood, or – and this is not at all rare – if the patient cannot report any reaction at all [23, 40]. In these cases, the BLA diagnosis can be withdrawn in a resource-saving manner even without testing (delabeling). It is not uncommon for the patient’s medical history to be uncertain or unreliable, and therefore a residual risk of a recurrence of an allergic reaction must be accepted with such a procedure; therefore, for the time being, the method is practiced as a precaution only in cases that subsequently receive the BL under direct medical supervision, not for an outpatient prescription. However, a severe reaction is very unlikely, as the individual should recall a previous anaphylactic or blistering reaction with emergency hospital treatment. 

## Risk-stratified approaches (application of algorithms) 

Predictive models that use clinical history as a basis to assess the true risk of β-lactam allergy in a patient can integrate all of the above strategies. Such diagnostic and treatment pathways (algorithms) have been developed and have already been tested in small numbers of patients [1, 12, 30, 40, 41]. The classification of the clinical manifestations of the patients into different risk groups, taken from the medical history, allows a differentiated approach depending on the present risk. If the patient’s history is appropriate, direct delabeling can be performed or direct provocation can be useful. This results in a good utilization of resources. Models have also been designed for use by non-allergists [42]. The rules for the courses of action must be created, standardized, and validated before they are applied in practice. 

## Discussion 

Verification of BLA diagnosis in a large number of patients is an important task in the antimicrobial stewardship program. With the large number of patients, this task cannot be accomplished by allergists alone. Patient safety when administering BL must be considered. However, it must also be remembered that this safety can never reach 100% because new sensitizations with reactions can also occur in any patient. In addition, the expected severity must be taken into account in the course of action. Thus, there is a decisive difference between taking a low risk for a mild reaction, e.g., an uncomplicated maculopapular exanthema, which frequently occurs in response to aminopenicillins, and for a severe, potentially life-threatening anaphylactic reaction. 

The patient’s medical history is essential for all delabeling procedures, especially for risk stratification. It has been shown that a history of uncomplicated childhood exanthema in the setting of an infection carries such a low risk of causing a non-severe reaction (exanthema) after administration of the same BL that direct provocation without prior skin testing is now recommended in the guidelines for children in this constellation [43]. After a maculopapular exanthema in adults in temporal connection with the intake of an aminopenicillin (amoxicillin, ampicillin), however, an allergy test is still recommended, which occasionally reveals sensitization; an exact percentage cannot be given, but is estimated by the authors at 10 – 20% [28]. All patients at high risk for a reaction and those at risk for a severe allergic reaction should continue to present to an allergist with classic (complete) allergy diagnostics. There are situations, such as severe bullous or exanthema with systemic involvement, cytotoxic or immune complex reactions, in which, without a specific need, no exposure to the trigger and administration of alternative antibiotics is recommended, because these reactions could not be safely managed after re-exposure. 

Among all strategies to verify a BLA diagnosis, consultation with an allergist with skin testing, laboratory testing if necessary, followed by provocation testing is the safest, but also the most time- and resource-consuming method [4, 28]. Multi-step fractionated provocation is the gold standard of allergy diagnosis. The number of provocation steps is not firmly standardized and can vary according to risk [44]. Skin testing and/or laboratory testing prior to BL administration under supervision reduces the risk of provocation testing to minimal to low, but requires trained personnel, a skin test-eligible patient, and appropriate resources [1, 45]. Many allergists unfortunately do not offer the most important, ultimate confirmation of a BLA by provocation test because they consider it too risky or have no opportunity for prolonged and safe monitoring. The few university clinics in Germany that offer this service alone do not have the capacity to test and to demonstrate tolerability for the large number of patients with often sparse evidence of BLA [46]. 

For the large number of patients with questionable BLA diagnoses, the health care system needs additional solutions, especially for cases with apparent low risk [3, 47]. The sensitivity of skin tests for immediate reactions has been reported to be as high as 97 – 99%, whereby a selection bias as well as a dependence on the time interval between reaction and diagnosis is evident [48]. Very severe immediate reactions can usually be excluded, if the skin test is negative. In settings with good monitoring and treatment readiness, such as intensive care units or emergency departments, there is a good infrastructure for drug provocation testing, if it is necessary and if the skin test is negative [49]. The risk for a positive reaction to the BL was low (< 5%), and mostly uncomplicated maculopapular exanthema occurred [50]. There are different reports of the value of skin testing depending on regional location. In Scandinavia, where penicillin G and penicillin V are predominantly given, a worse negative predictive value is reported than in Central and Southern Europe, where more broad-spectrum BLs, such as aminopenicillins, are used [51]. A disadvantage of skin testing without provocation is that trained personnel must also be available for skin testing, and testing is time consuming. 

In patients at low risk of non-severe reactions, direct provocation testing may be useful to rule out BLA (whereas it should never be done to confirm a physician’s suspicion!). This method is low resource-intensive but involves a higher residual risk of reactions than after skin testing. Provocation usually has good negative predictive value. However, the duration and dosage of provocation remains controversial among experts. While the majority of allergy centers consider a 1-day or 2-day provocation with reaching the usual single dose or full-day dose sufficient, individual centers propagate the administration of BL of up to 7 days [47, 52]. So far, guidelines recommend direct provocation only in children after the development of uncomplicated exanthema [43]. Two systematic reviews also examined the safety and efficacy of direct oral provocation by allergists and non-allergists in adults [33, 34]. However, data for use in immediate reactions are still insufficient. 

In some cases, the patient’s history alone may be sufficient to rule out BLA with reasonable certainty [23, 40]. In Germany, a comparatively simple algorithm with five steps or sets of questions has been used and has been tested in this regard [23]. First results confirm e.g. that, after a “rash” in childhood in connection with the intake of a penicillin, a BLA is extremely improbable [13, 43, 53]. However, many patients are rock-solidly convinced of their BLA and find it difficult to accept any assessment to the contrary just because of their history. The possibility of error and limited informative value of the history, which is criticized when applying standardized questioning, also applies in principle to every allergy test and every other delabeling procedure, because e.g. the selection of the test method or the classification of the cases into so-called risk groups is also primarily based on the history. 

Clinical risk-stratified treatment pathways (algorithms) for drug allergy, once validated, will become established in the future as a sufficiently safe and effective method for clarification in patients with a designated BLA. Classification of patients’ clinical manifestations into different risk groups reduces the need for skin testing and/or comprehensive allergy counseling, allows direct administration under supervision with or without fractionated provocation (test dosing), and thus saves resources [1, 3, 40]. Administration of non-cross-reactive BLs significantly increases safety, but does not allow “delabeling” and is best suited for emergency situations and inpatients [1, 22]. Patients at no risk can be delabeled directly. For those with “low risk”, different strategies are currently under review. However, the definition of “low risk” currently remains the subject of controversy. Some important issues and challenges in withdrawing a β-lactam allergy label are summarized in Table 5. Validation efforts in high numbers of patients in a multi-center approach for different models are needed before they are used in daily practice. Evidence suggests that experience and decision support training may be needed to use such models correctly. Computerized and systematic assessment tools have been developed and are being tested. 

It remains to be noted that in parallel to the strategies for delabeling, better education and training of the medical profession with regard to drug reactions should be demanded. The knowledge alone that only a very small proportion of acute skin reactions associated with a febrile infectious disease and the use of a BL antibiotic are expressions of a BLA could ensure that the number of hastily declared BLAs is reduced [7, 21, 54]. 

## Conclusion 

The diagnosis of BLA is widespread, often questionable, and can lead to less effective treatments with second-line antibiotics as well as negative public health consequences such as antibiotic resistance, high resource use, and costs. Clinical history has long been the essential component of BLA evaluation. Removal of the diagnosis of BLA as an aspect of antibiotic stewardship programs is becoming more of a focus. There is a need for validated approaches that optimally combine the use of history and risk stratification with validated allergy testing procedures. In addition to classical routine allergy diagnostics, evidence-based prediction strategies are very promising, but differ in crucial areas such as the populations studied, the predictor variables used, and the accuracy achieved, and thus can negatively affect patient safety. These risk stratification strategies are currently under investigation and pave the way for future large-scale multi-center studies. 

## Funding 

No funding. 

## Conflict of interest 

No conflict of interest for the topic of this paper. 

**Table 1.** Common reasons for mislabeling a patient as β-lactam allergic.

Misinterpretation of
– known predictable side effects as allergy (e.g., pure gastrointestinal symptoms due to alteration of the intestinal microbiome)
– infection-induced urticaria as an immediate-type drug reaction
– infection-induced viral exanthema as drug exanthema
– non-specific symptoms or somatoform reactions as allergy
– known reactions in the family as an indication of β-lactam allergy
– a fear of an allergy as an actual allergy (“I react to all medications”)

**Table 2.** Consequences of falsely labeling of patients as β-lactam allergic for the antimicrobial stewardship program.

– Prescription of less suitable antibiotics (“second choice”)
– Delay in antibiotic therapy
– Higher healthcare costs
– Greater prevalence of antibiotic resistant germs
– More frequent treatment failures
– Longer hospital stays
– Higher mortality risk
– More frequent treatment in intensive care

**Table 3.** Medical history-based risk stratification for suspected diagnosis of β-lactam allergy.

1. No evidence of an unexpected β-lactam hypersensitivity reaction
– Gastrointestinal reaction only (e.g., nausea, vomiting, diarrhea)
– Only nonspecific reaction (e.g., headache, rhinoconjunctivitis, palpitations), often associated with fear of drug hypersensitivity
– Urticaria with onset > 1 day after discontinuation of β-lactam or persisting for days after drug discontinuation
– Exanthem with onset > 1 week after discontinuation of β-lactam
– Only family history positive for drug hypersensitivity
2. Indications of questionable reactions with low risk
– Urticaria occurring only after a delay (> 6 hours after ingestion)
– Non-remembered reaction > 10 years ago without therapy
– Mild rash in childhood, especially associated with infection
3. Evidence of non-severe delayed-onset drug exanthema
– Maculopapular (uncomplicated) drug-induced exanthema with therapy < 10 years ago
4. Indications of moderately severe immediate reactions
– Urticaria
– Angioedema
– Tachycardia
5. Evidence of severe drug reactions with high risk
– Vomiting, diarrhea along with other anaphylaxis symptoms
– Wheezing / dyspnea
– Blood pressure drop
– Unconsciousness
– Anaphylaxis
– Cardiovascular and/or respiratory arrest
6. Indications of possible severe β-lactam hypersensitivity reactions that cannot be treated with sufficient safety in case of recurrence and therefore usually leads to an elimination of β-lactams and administration of alternative antibiotics
– Drug reaction with eosinophilia and systemic symptoms (DRESS, drug hypersensitivity syndrome)
– Hemolytic anemia or cytopenia
– Acute nephritis or hepatitis
– Serum sickness
– Severe exanthema with blistering of the skin and/or mucosa (Stevens-Johnson syndrome, toxic epidermal necrolysis)

**Table 4.** Comparison of strategies to remove spurious β-lactam allergy labels (adapted from [3]).

Strategy	Methodology	Advantages	Disadvantages
Classic allergy diagnostics	H, ST, LT, PT	Highest safety, proven procedure, allergists, highest risk reduction for immediate reactions before PT, good NPV, cross-reactivities can be tested	High cost, resource- and time-intensive, too few testing options for affected patients, validity of laboratory testing insufficiently verified
Skin testing	ST	Minimizes risk for severe reaction, risk low for all immediate reactions, moderate risk reduction for exanthema	Skin test-negative exanthema and immediate reactions after testing not excluded with certainty, different significance of skin testing for different populations and β-lactam classes
Direct provocation	PT	Good NPV, well-tested in childhood in patients at low risk of non-severe reactions (e.g., uncomplicated exanthema in childhood infection), not resource-intensive	Risk for reactions higher, few data for use in adult exanthema, insufficient data for use in immediate reactions.
Standardized questioning (consideration of the medical history alone)	H	Majority of patients interviewed are not allergic, sometimes clear statements can be derived from H alone, resource-conserving	Residual higher risk has to be accepted, not very convincing for the patient, administration of the β-lactam only under direct medical supervision (as a measure for risk minimization)
Risk stratified approaches (application of algorithms)	Variable, depending on H	Different approaches depending on the H of the patient, therefore combines different strategies, good utilization of resources.	Complex courses of action that require clear rules, possibility of errors, validation so far only by limited observatory trials

H = history, ST = skin tests, LT = laboratory tests, PT = provocation tests, NPV = negative predictive value.

**Table 5.** Problems and challenges in the withdrawal of a β-lactam allergy label.

– Too few allergists with experience in this field
– Rare allergological testing in children
– Patients avoid β-lactams despite withdrawal of suspected diagnosis
– Duration and conditions of provocation testing are not standardised
– Laboratory tests for the detection of β-lactam allergy not sufficiently validated
– Skin testing is time-consuming, cost-intensive, and β-lactams are largely not approved for this indication (skin testing)
– Cross-reactivities between β-lactam antibiotics not fully known
– Regional differences in the triggering β-lactams and diagnostic procedures in Europe.
